# Are pfhrp2 and pfhrp3 Deletions a Concern in Latin America? A Critical Review

**DOI:** 10.3390/pathogens15040368

**Published:** 2026-03-31

**Authors:** Beatriz Pires da Silva, Pablo Secato Fontoura, Cláudio Tadeu Daniel-Ribeiro, Maria de Fátima Ferreira-da-Cruz

**Affiliations:** 1 Laboratório de Pesquisa em Malária, Instituto Oswaldo Cruz, Fundação Oswaldo Cruz (Fiocruz), Rio de Janeiro 21041-361, Brazil; beatrizsilva@aluno.fiocruz.br (B.P.d.S.); ribeiro@ioc.fiocruz.br (C.T.D.-R.); 2 Centro de Pesquisa, Diagnóstico e Treinamento em Malária (CPD-Mal), Reference Malaria Laboratory of the Extra-Amazonian Region for the Brazilian Ministry of Health, Secretaria de Vigilância Sanitária & Fiocruz, Rio de Janeiro 21041-361, Brazil; 3 Coordenação Geral de Eliminação da Malária—CGEMA, Secretaria de Vigilância em Saúde e Ambiente, Ministério da Saúde, Distrito Federal, Brasília 70058-900, Brazil; pablo.fontoura@saude.gov.br

**Keywords:** malaria, *Plasmodium falciparum*, malaria RDTs, HRP2 deletions, Latin America

## Abstract

*Plasmodium falciparum* with deletions in the *pfhrp2* and *pfhrp3* genes has increasingly challenged national malaria control efforts worldwide by reducing the reliability of HRP2-based rapid diagnostic tests. This review explores available reports and evidence on this issue across Latin America. The results show a varied distribution, with countries such as Peru, Colombia, and Brazil reporting the emergence and spread of multidrug-resistant strains. Meanwhile, Caribbean countries and parts of Central America report low prevalence or no deletions. Understanding these patterns is essential for adjusting surveillance strategies, especially in countries nearing malaria elimination.

## 1. Introduction

Malaria is an acute febrile infectious disease caused by protozoan parasites of the genus *Plasmodium*. It is transmitted through the bite of an infected female *Anopheles mosquito* and, in humans, is characterized by symptoms such as fever, chills, and headache [1,2].

Considered the parasitic disease with the greatest impact worldwide, in 2023, approximately 263 million cases were reported globally, resulting in 597,000 deaths, with 95% of these deaths occurring in Africa. In the Americas, there are 17 endemic countries. Overall, there were around 548,000 cases and 342 deaths in 2023 [3].

Eight species of *Plasmodium* are known to infect humans: *P. malariae* (Laveran, 1881); *P. vivax* (Grassi; Feletti, 1890); *P. falciparum* (Welch, 1897); *P. ovale* (Stephens, 1922), now considered two species, *P. ovale curtisi* and *P. ovale wallikeri* (Sutherland et al., 2010; Snounou, 2024); *P. knowlesi* (Sharp et al., 2020); *P. cynomolgi* (Ta et al., 2014); and *P. simium* (Da Fonseca, 1951) [4,5,6,7]; the last three are zoonotic [8]. Among these species, *P. vivax* and *P. falciparum* cause the majority of cases worldwide. In the Americas, 73% of cases are caused by *P. vivax*, 27% by *P. falciparum*, and 0.5% by mixed infections [3].

*P. falciparum* is the deadliest species, mainly causing deaths in children under 5 and pregnant women in Africa. Due to its potential for severe illness, the species has high rates of sickness and death if not diagnosed quickly. Therefore, fast and accurate diagnosis is essential for patients suspected of having the disease [9].

The diagnostic method considers microscopy the gold standard, enabling the identification of species and parasite density [2]. However, it requires significant expertise and resources from operators, which can be difficult to maintain in challenging environments often endemic to the disease. The use of rapid diagnostic tests (RDTs) has revolutionized malaria diagnosis by needing minimal infrastructure and training, and providing results in about 15 min. Consequently, RDTs can be employed in fieldwork and health centers located in remote areas [10].

Approximately 350 million RDTs were distributed by national malaria control programs in 2023, emphasizing the global significance of this method [3]. These tests use immunochromatography to detect proteins released by the parasite. Tests that identify the Histidine-Rich Protein 2 (HRP2) antigen are often preferred because of their higher sensitivity [11].

HRP2 is a *P. falciparum*-specific protein that is abundantly secreted during the blood stages and contains epitopes that elicit antibody responses, which is why it is used in many tests. HRP2-based tests are more sensitive than pLDH-based tests. Cross-reactions may occur between the epitopes of HRP2 and HRP3, another *P. falciparum*-specific protein that has been less studied than HRP2. The genes encoding these proteins have two exons, exon 1 and exon 2. Exon 1 and the start of exon 2 contain signal and cleavage sequences, while exon 2 includes the highly polymorphic coding sequences [12].

Since 2010, antigen deletions have led to false-negative RDT results. The earliest reports of this issue in the literature originate from Latin America. Currently, *pfhrp2* and *pfhrp3* deletions have been documented in 41 endemic countries across Africa, Asia, and the Middle East [3,13]. Because of this problem, the WHO has set a threshold: when the prevalence of patients with strains carrying *pfhrp2*/*3* deletions causing false-negative RDT results exceeds 5%, it is recommended to use RDTs targeting other *Plasmodium* antigens [3].

Because of their impact on diagnostic accuracy, it is important to understand the regional context where these deletions were first identified and tracked.

Latin America is essential for understanding these deletions, not only because it has endemic areas of the disease but also because the first case reported globally was identified in Peru [9]. Since then, countries in the region have conducted targeted investigations into the prevalence of these genetic variants, highlighting the need for expanded molecular surveillance.

Despite the increasing global research on such deletions, a notable gap exists in Latin America, not only in the number of studies conducted but also in the consistent use of protocols for tracking deletions.

Therefore, the goal of this narrative review is to synthesize the available evidence on this topic, focusing on molecular surveillance and epidemiological interpretations; examine methodological differences; discuss the implications of these findings; and compare results across countries in the region.

## 2. Materials and Methods

This narrative review summarizes and compares reports of *pfhrp2* and *pfhrp3* deletions across Latin America. A structured literature search was conducted in PubMed, SciELO, and Scopus, including papers published between 2010 and January 2026. The following keywords and Boolean operators were used: (“*P. falciparum*” AND (*pfhrp2* OR *pfhrp3*) AND deletion AND the name of each country) and (“*Plasmodium falciparum*” AND (*pfhrp2* OR *pfhrp3*) AND deletion AND the name of each country); 29 papers met the inclusion criteria.

The selection of studies was based on predefined inclusion and exclusion criteria, ensuring that the selected works included original research articles on molecular surveillance of deletions. Review articles, case reports, and protocols for standardizing molecular methods were excluded.

To reduce potential duplication bias, the articles were carefully examined for cohort overlap, comparing author groups, study designs, sample sources, recruitment centers, and study periods. In cases of suspected overlap, methodological descriptions were compared to confirm the dataset’s independence. Based on this assessment, it was determined that all included studies represented distinct study populations, and no duplicate cohorts were included in the paper.

Studies were classified as convenience samples when they originated from routine diagnostic collections without population-based recruitment. Regional malaria prevalence estimates were not derived from the included studies but were based on epidemiological data reported by the World Health Organization (WHO). These estimates were included to provide a standardized epidemiological context for each eco-region and to facilitate comparisons of deletion patterns across different transmission settings.

The data were arranged by country and region to facilitate comparative, descriptive, and temporal analyses.

## 3. Results

### 3.1. An Overview by Country

#### 3.1.1. Peru

The first global report of these deletions came from Peru in 2010, during investigations in the Peruvian Amazon. A retrospective study analyzed 148 samples with false-negative results from 2003 to 2007. Of these, 41% and 70% lacked the *pfhrp2* and *pfhrp3* genes, respectively, while 21.6% lacked both genes [13]. This study was the first to report deletions in the *pfhrp2* and *pfhrp3* genes and link them to false-negative RDT results. This discovery underscored the urgent need to update diagnostic strategies in this region.

The discovery prompted further research both nationally and across Latin America, particularly in areas with ongoing transmission.

In the same year, an evaluation of the RDT Parascreen™ was carried out in the Iquitos region of the Peruvian Amazon. The test detects HRP2 and pLDH across all *Plasmodium* species. To assess its performance, samples from 332 febrile patients suspected of having malaria were tested between October and December 2006. The RDT results were compared with microscopy and PCR [14]. The test demonstrated low sensitivity, especially for *P. falciparum*, leading to many false negatives and increasing the risk of severe disease. Its sensitivity compared to microscopy was 53.5% [14]. The authors attributed the poor performance to low parasite densities and the presence of parasites with deletions. Notably, the same test yielded different results in various countries, with sensitivity ranging from 94% in Kenya to 96.3% in India [15,16]. In Ethiopia, the results resembled those in Peru [17]. In fact, an evaluation conducted by the World Health Organization (WHO) RDT program in collaboration with the Special Programme for Research and Training in Tropical Diseases (TDR), Foundation for Innovative New Diagnostics (FIND), and Centers for Disease Control and Prevention (CDC) showed the same test having satisfactory detection performance. The test successfully identified the species across multiple testing rounds using cultured parasites, patient-derived parasites, and negative samples [18].

Years later, a study was conducted to determine the origin and explore possible reasons for the geographic spread of the deleted parasites in the Peruvian Amazon. To this end, samples from different time periods were analyzed using microsatellite markers to genotype the strains. The researchers concluded that the *pfhrp2* deletions originated in at least two ancestral lineages, indicating multiple sources rather than a single isolated event. They also found that the population of parasites with deletions increased over time, by roughly 20–40% over seven years, with significant implications for the effectiveness of HRP2-based RDTs. The authors noted that it remains unclear whether any biological selection process favored this expansion in the region and whether it is still ongoing [19].

Similarly, in 2015, an investigation in the Tumbes region traced the origins of an outbreak that occurred five years earlier, during which no *P. falciparum* malaria cases had been reported since 2006. The study also genotyped the samples using microsatellite markers to determine genetic relationships among the isolates. In this case, all isolates shared the same genotype, indicating a single source, and showed deletions in the *pfhrp2* and *pfhrp3* genes, as well as mutations in genes linked to chloroquine and sulfadoxine/pyrimethamine resistance [20]. Interestingly, samples collected from an area far from the Amazon shared the same genotype as those from Loreto in 2010, which is located in the Amazon basin, suggesting that parasites had been introduced through human movement along river and road networks [13,20]. This finding underscores the critical role of population mobility in the spread of deletion-carrying parasites, as migration helps introduce strains into new areas and could weaken the effectiveness of local diagnostic strategies. The study highlights that an apparently isolated outbreak may result from the importation of resistant strains with significant genetic deletions, underscoring the need for effective surveillance and for adapting diagnostic methods during disease reintroductions [20].

In 2013, another outbreak occurred in a different part of Peru, linked to parasites imported from Loreto, a city in the Peruvian Amazon. This outbreak took place in Cusco, in the southeastern Andean region, where no *P. falciparum* cases had been reported since 1946. The researchers compared these isolates with the Bv1 clone, which caused an outbreak in Tumbes, a northern coastal region, between 2010 and 2012 [20,21]. The results showed that the Cusco isolates matched this clone. The BV1 clone is especially important because it is multidrug-resistant and has *pfhrp2*/*3* deletions, which are concerning because they may hinder both malaria diagnosis and treatment. The investigators concluded that the outbreak was caused by two construction workers who traveled from Loreto to Cusco in 2013, showing that the parasite was introduced through human movement. The Cusco outbreak was the second in Peru caused by the same clone, highlighting its regional spread and ability to establish transmission in areas previously free of malaria [21].

In a 2021 genomic study of *P. falciparum* from the Peruvian Amazon and northern coast, researchers identified three subpopulations: Bv1, Clonet D, and Acre-Loreto type. Bv1 was the most common lineage, occurring in both regions, whereas the others were limited to the Amazon. Bv1 lacked *pfhrp2* and *pfhrp3* coverage across all analyzed isolates and exhibited a high prevalence of resistance-associated mutations, indicating a dual threat of diagnostic escape and drug resistance. Additionally, four *PfS47* haplotypes, a gene involved in immune evasion in *Anopheles* mosquitoes, were identified. Bv1 carried unique haplotypes, with one confined to Loreto and another (haplotype type 1) shared between Loreto and Tumbes. The Brazilian *PfS47* haplotype closely resembled the Peruvian type 1, supporting the idea of introduction from Brazil. Population genomics also revealed a clonal expansion and likely replacement by Bv1 since 2011, as well as gene flow between Loreto and Tumbes through shared *PfS47* haplotypes, consistent with geographic spread facilitated by human movement. These findings highlight that deletion-carrying lineages can become epidemiologically dominant and spread across regions, especially in border areas with transnational circulation, increasing the risk of false-negative results in PfHRP2-based RDTs. The coexistence of chemoresistance and immune evasion mechanisms suggests that this lineage may confer adaptive advantages that help its persistence and expansion [22].

The following year, an analysis was performed on the prevalence, geographic distribution, and temporal changes of *pfhrp2* and *pfhrp3* deletions in the Peruvian Amazon. The authors discovered that 67% of the tested samples showed double deletions in *pfhrp2* and *pfhrp3*. The percentage of deleted strains increased from about 14% in 2011 to roughly 88% in 2016, then leveled off at approximately 65% in 2018, indicating rapid growth followed by a plateau of these lineages. This rise was linked to the dominance of the H8 haplotype, which seemed to replace other local lineages. These findings support the hypothesis that this haplotype may have a selective adaptive advantage [23]. Notably, the H8 haplotype was closely related to the previously identified Bv1 strain, although it was not identical [21,22,23].

In the same year, the patterns and frequencies of *pfhrp2* and *pfhrp3* deletions were examined in 93 *P. falciparum* samples collected from the Peruvian Amazon between 2009 and 2010. They found that approximately 35% of samples had a deletion in *pfhrp2* and about 57% had a deletion in *pfhrp3*. Eight distinct lineages were identified, with Bv1 being the most common among isolates with deletions. Notably, all Bv1 isolates showed double deletions in both *pfhrp2* and *pfhrp3*, emphasizing the risk of false-negative results in PfHRP2-based rapid diagnostic tests. Other lineages, such as V4, also carried deletions, indicating that multiple genotypes capable of evading diagnosis were circulating in the region at that time [24].

In 2024, a study was conducted in an indigenous community in Nueva Jerusalén, a region with high population mobility, to investigate transmission patterns of *P. falciparum* and *P. vivax* and to assess the prevalence of *pfhrp2*/*3* deletions and resistance markers in *P. falciparum*. The study found that 32–50% of *P. falciparum* isolates had deletions in *pfhrp2* and/or *pfhrp3*, highlighting the risk of false-negative results in HRP2-based rapid diagnostic tests. The study also identified polymorphisms associated with antimalarial resistance, and the authors stressed that population mobility likely facilitates the spread of parasites carrying deletions, creating challenges for malaria surveillance and control [25].

Among these Peruvian studies, the vast majority were retrospective and often focused on regions where outbreaks occurred. The main focus was on the Peruvian Amazon, especially the department of Loreto, as well as on the regions of Tumbes and Cusco, located in the northwest and southeast, respectively. Based on microsatellite genotyping analyses conducted by six of the nine studies, haplotype Bv1 was notably present between 2006 and 2011, followed by the predominance of haplotype H8, both of which have double deletions and mutations that confer resistance to antimalarial drugs.

Another interesting point is that heterogeneity was observed among the included studies regarding the molecular protocols used, including differences in genetic targets and criteria for confirming genetic deletions. However, the reported prevalence estimates and overall patterns were consistent across studies, suggesting that methodological variability did not significantly impact the overall results.

According to the 2024 World Malaria Report, Peru reported about 29,000 malaria cases in 2023, reflecting an 18% decrease from the previous year [3]. Transmission mainly remains concentrated in the Amazon basin, where high population mobility linked to mining and logging promotes parasite circulation across border regions, especially those shared between neighboring countries. These patterns could facilitate the spread of deletion-carrying strains, increasing the risk of underdiagnosis and complicating control efforts [26].

#### 3.1.2. Colombia

The detection of *pfhrp2*- and *pfhrp3*-negative isolates in the Peruvian and Brazilian Amazon regions bordering Colombia prompted researchers to investigate this issue further. In a 2015 study, 100 historical samples collected between 1999 and 2009 from six Colombian regions were analyzed using PCR and microsatellite markers. Overall, 18% of individuals showed deletions in the *pfhrp2* gene, primarily from the Amazonas Department, which borders Peru and Brazil. In contrast, 52% exhibited deletions in the *pfhrp3* gene, distributed across the country. The population structure analysis identified four genetic groups, with most *pfhrp2*-deleted parasites concentrated in a single Amazonas group. Additionally, the authors examined the flanking genes of *pfhrp2* and *pfhrp3* and found frequent deletions, indicating broader chromosomal rearrangements [27]. These findings reinforce concerns that HRP2-based rapid diagnostic tests could be significantly affected in Colombian endemic areas, especially in the Amazon region.

Years later, the same group conducted a broader genetic study to explore the origins and traits of parasites linked to a 2013 *P. falciparum* outbreak. They analyzed 365 samples collected from 2003 to 2012 across seven Colombian departments, using PCR of *pfhrp2*/*3* and neighboring genes, along with microsatellite analysis. In Amazonas, 38% of isolates lacked *pfhrp2*, and all of these also lacked *pfhrp3*. Throughout the country, 43% of parasites showed deletions in *pfhrp3*.

Notably, all parasites lacking *pfhrp2* and/or *pfhrp3* also had deletions in one or both flanking genes, indicating large chromosomal rearrangements. Population structure analysis revealed that 93% of *pfhrp2*-negative isolates clustered within the same haplotype, matching the multiresistant clone Bv1, the same lineage responsible for outbreaks in Peru [21,22,23,28]. Other minor clonal lineages (EV1 and F) were also identified. Furthermore, analysis of HRP2 sequences in non-deleted isolates identified 17 unique repeat variants, further undermining the effectiveness of RDTs. These findings demonstrate that *pfhrp2*/*3*-deleted and multiresistant parasites have existed in Colombia for years, especially in the Amazon border region, highlighting the cross-border movement of high-risk strains and the urgent need for alternative diagnostic methods [28].

Researchers studied the spatial and temporal patterns of *P. falciparum* transmission in Guapi, a location on the Pacific Coast. They created operational transmission units to evaluate connectivity within the parasite population, estimate the size of asymptomatic reservoirs, and identify deletions in the *pfhrp2*/*pfhrp3* genes. Testing 31 samples from symptomatic patients, they found 6.2% with a *pfhrp2* deletion, with no deletions in neighboring genes. In contrast, 87.1% had *pfhrp3* deletions, and most also had deletions in nearby genes. Many samples also showed mutations linked to antimalarial resistance. Microsatellite analysis identified three subpopulations (A, B, and C) circulating in Guapi and nearby rural areas. Notably, deleted strains appeared across these subpopulations, indicating that this is not an isolated event. These findings emphasize that, even in coastal regions far from the Amazon Basin, deleted strains are present and highlight the broader geographic spread, raising concerns about how these deletions might impact HRP2-based rapid diagnostic tests [29].

Recently, Olivera et al. conducted a cross-sectional, multi-site study in five malaria-endemic municipalities along the Colombian Pacific Coast, following the WHO protocol for *pfhrp*/*pfhrp3* deletion surveillance, as previous studies in the country had used different protocols [30,31]. In fact, the WHO protocol aims to standardize surveillance methodologies across studies to estimate the prevalence of false-negative RDT results due to *pfhrp2*/*3* deletions [31].

Among 347 patients, 4.61% (16/347) had false-negative results on HRP2-based RDTs. Molecular tests revealed that all false-negative samples contained the *pfhrp2* gene, although two had partial deletions, each causing a 100 bp reduction in exon 2. Conversely, 93.8% (15/16) of these samples had a complete deletion of the *pfhrp3* gene. The authors noted that, despite the low false-negative rate associated with *pfhrp2* deletion, RDTs remained reliable in those regions and highlighted the importance of ongoing surveillance in Colombia. Across the cited studies, most investigations employed retrospective designs. Although most studies were conducted across multiple regions, the highest prevalence of deletions was observed in the Amazonas department, which borders Peru and Brazil. Still, as mentioned earlier, the last two studies report risk along the Pacific coast, indicating the deletion’s spread throughout the country.

Among the four studies mentioned, two used the same non-WHO molecular protocol [27,28], one combined this protocol with the WHO-recommended method [29], and one followed the WHO protocol exclusively [30].

Although molecular protocols vary across studies, the magnitude and consistent increase over time in *pfhrp3* deletion prevalence, from 43% to 87% and then to 93.8%, suggest that this pattern is unlikely to be explained solely by methodological differences. This steady rise over nearly twenty years, especially in the Pacific region, indicates probable clonal expansion and the growing presence of the deletion in the parasite population. While methodological differences should be considered when interpreting the exact figures, the fact that adopting the WHO-recommended protocol later on did not reduce these rates suggests we are observing a true epidemiological change rather than a technical artifact.

Malaria epidemiology in Colombia varies across regions, with transmission mainly happening in rural areas of the Amazon and Pacific regions. Although *P. vivax* is most common, occasional *P. falciparum* outbreaks still occur. About two-thirds of municipalities have ecological and epidemiological conditions that are suitable for transmission, and human movement helps maintain parasite circulation. Colombia has aimed to eliminate malaria by 2030 [32].

#### 3.1.3. Brazil

To assess the extent of *pfhrp2* and *pfhrp3* deletions in the Brazilian Amazon, researchers analyzed 198 *P. falciparum* samples from previous surveys across six sites in three states: Acre, Rondônia, and Pará. Samples from Acre were collected in Cruzeiro do Sul. Samples from Rondônia were obtained in Monte Negro, and samples from Pará were collected at two locations: Goianésia do Pará and Itaituba. The study found significant regional differences in *pfhrp2* deletion rates: 31.2% of isolates from Acre had *pfhrp2* deletions, compared with only 3.3% from Rondônia and none from Pará. In contrast, *pfhrp3* deletions were more frequent, with rates ranging from 18.3% to 50.9% across states. These findings highlight geographic variation in deletion prevalence and underscore the need to reconsider the use of HRP2-based rapid diagnostic tests in some areas, as well as the importance of ongoing molecular surveillance to support malaria control efforts in the Brazilian Amazon [33].

Later, the frequency of *pfhrp2* and *pfhrp3* deletions in isolates from endemic regions of the Brazilian Amazon was studied through a retrospective, observational, cross-sectional approach. The authors reexamined isolates from patients in the municipalities of Cruzeiro do Sul, Acre, and Manaus, Amazonas, between 2016 and 2017. The samples were tested using PCR targeting exons 1–2 and 2 of both genes. The study found high deletion rates: 72% of isolates from Acre (71/99) and 100% from Manaus (60/60) showed *pfhrp2* deletions, while *pfhrp3* deletions were detected in 95% and 98% of isolates, respectively. Overall, nearly 80% of the samples had deletions in one or both genes. Due to this high prevalence, the authors highlighted the need to reassess the use of HRP2-based rapid diagnostic tests in these areas, as such deletions can hinder malaria diagnosis and elimination efforts, emphasizing the importance of alternative diagnostic methods and continued molecular surveillance [34].

Subsequently, another study investigated the genetic diversity of the *pfhrp2* gene in the Brazilian Amazon. A total of 132 samples collected from 2002 to 2020 across four states (Amapá, Mato Grosso, Rondônia, and Roraima) were analyzed. The study found that 10% of isolates had deletions in the *pfhrp2* gene; however, this notable prevalence did not significantly impact the performance of HRP2-based rapid diagnostic tests, likely due to polyclonal infections, as indicated by microsatellite genotyping. The research also observed a substantial discrepancy in deletion detection between conventional and nested PCR protocols, highlighting the need for rigorous molecular workflows in deletion assessment. These findings reinforced the genetic diversity in the Brazilian Amazon and pointed out methodological challenges in molecular surveillance. Additionally, the researchers identified 10 distinct HRP2 amino acid repeat sequence patterns, each unique to one of the studied regions, and noted that these patterns resembled those previously reported in French Guiana [35,36].

Similarly, Bally et al. conducted a descriptive observational study in the Middle Rio Negro region of the Brazilian Amazon, specifically in the municipality of Barcelos, Amazonas. In this endemic area, deletion studies had not been performed previously. The researchers analyzed *P. falciparum* samples collected between 2003 and 2016 from various cross-sectional studies. Of the 82 samples, 52.4% (43/82) showed *pfhrp2* deletions (34.2% with only *pfhrp2* deleted and 18.3% with both *pfhrp2* and *pfhrp3* deletions), while 41.5% (34/82) exhibited *pfhrp3* deletions (23.2% with only *pfhrp3* deleted). Notably, parasites with double deletions (*pfhrp2*/*3*) were more common among asymptomatic individuals, suggesting a potential link between deletion status and clinical presentation. Given the high prevalence of these deletions, the authors emphasize the importance of ongoing molecular surveillance in this region [37].

In the same way, a molecular surveillance study was carried out in the tri-border region of Brazil, Venezuela, and Guyana. The study examined 365 *P. falciparum* samples collected from 2016 to 2018 using microscopy, RDTs, and molecular methods, including nPCR, qPCR, and immunological assays. By combining these approaches, the study found a very low prevalence (1%) of *pfhrp2*-deleted parasites, confirmed by the absence of HRP2 detection. In contrast, although some isolates failed to amplify *pfhrp3*, the study did not report a high-confidence deletion rate for this gene. Notably, most false-negative RDT results were associated with low parasitemia [38]. The genetic diversity analysis of both studies revealed low diversity and a *pfhrp2* sequence pattern similar to those previously described in French Guiana [35,38]. It is important to recognize differences in deletion detection methods and in the prevalence of *pfhrp2* deletions, despite some geographic overlap between the studies. The influence of different time periods and the large influx of Venezuelan migrants during sample collection could account for these seemingly conflicting findings [36,38].

Regarding case prevalence, 70% of samples came from men in mining areas. Of these, 92.6% were from Venezuela and 4.9% from Guyana. Given the high population mobility in this border region, the authors stress the importance of ongoing molecular surveillance to track the potential spread of *pfhrp2* deletions, even though the current low prevalence remains below the WHO-recommended threshold for changing diagnostic strategies [38].

All the studies conducted in Brazil were retrospective, with some focusing on areas with high border mobility.

A sharp increase in the prevalence of the *pfhrp2* deletion was observed in Acre, rising from 31% (2012) to 71% (2016–2017) and reaching 100% in Manaus during the same period. While the previous study employed a molecular protocol not recommended by the WHO, the subsequent investigation used only the WHO protocol. Despite this methodological difference, the progressive increase in prevalence may suggest that the observed trend cannot likely be explained solely by the variation in protocol [33,34].

However, considerable variability in prevalence estimates was observed when different molecular approaches were directly compared within the same study (46% vs. 10%), with the WHO protocol reporting a higher prevalence and the nested protocol a lower prevalence, demonstrating that protocol selection significantly affects the detection of deletions [36].

Similarly, Mascarenhas et al. (2025) reported that the lower number of *pfhrp2*-negative samples identified by multiplex qPCR compared with nested PCR suggests that methodological differences, including assay sensitivity and amplification thresholds, can significantly affect prevalence estimates [38]. This finding further emphasizes the need for caution when comparing deletion frequencies between studies using different molecular platforms. Therefore, although the temporal trend indicates clonal expansion and possible fixation in certain regions, caution is advised when comparing absolute prevalence values across studies with different molecular strategies.

In Brazil, malaria transmission is mainly concentrated in the Amazon region, which accounts for over 99% of indigenous cases [39]. In this setting, rapid diagnostic tests (RDTs) have a vital operational role, especially in remote indigenous and riverside communities where laboratory infrastructure is limited. In these areas, RDTs are often the main diagnostic tool.

#### 3.1.4. Ecuador

Between 2012 and 2013, an outbreak of *P. falciparum* malaria took place in Esmeraldas, a region along the Pacific coast. Over 100 cases were reported during this time, compared to the low incidence seen in previous years.

To examine the source of this outbreak and help prevent future cases, researchers analyzed parasite isolates using neutral microsatellites, *pfhrp2* genotyping, and molecular markers associated with antimalarial resistance (*pfcrt*, *pfmdr1*, *pfdhfr*, and *pfdhps*). Results indicated that 96% of samples belonged to a single clonal lineage previously reported along Peru’s northern coast. Nearly all isolates carried resistance-related genotypes, and 97% had an intact *pfhrp2* gene. These findings imply that the outbreak likely originated from an external source, likely Peru, and that HRP2-based RDTs will remain effective for detecting this strain [40].

Only one eligible Ecuadorian study was identified, which limits the ability to compare it with other studies. The available study used a retrospective design and did not follow the WHO-recommended molecular protocol for deletion surveillance. Although the results indicate deletions in this context, the lack of a standardized methodology and additional studies prevents assessment of trends over time, methodological consistency, or regional differences. As a result, the evidence for this region remains limited, and any conclusions about transmission dynamics or implications for diagnostic policy should be approached with caution.

Malaria transmission in Ecuador is mainly limited to Amazonian provinces bordering Peru and Colombia. Ongoing government investment in control programs has significantly reduced malaria cases, positioning the country as a strong candidate for elimination in the region [41,42].

#### 3.1.5. Suriname

In response to reported *pfhrp2*/*3* deletions across South America, a 2015 study in Suriname evaluated the local situation. Among 78 samples analyzed, 14% showed deletions in the *pfhrp2* gene and 4% in *pfhrp3*. Some deletions also extended into neighboring genes, indicating larger genomic deletions. Population structure analysis revealed no dominant genetic cluster, and *pfhrp2*-negative isolates did not seem to originate from a single clone [43].

In this country, only one study met the inclusion criteria, and it was conducted before the disease was eliminated nationally. After elimination, routine surveillance and diagnostic demand likely decreased, which may explain the lack of additional studies. Consequently, the available evidence is insufficient to guide current decisions on diagnostic policies, although the study offers valuable baseline data on the prevalence of deletion before transmission was halted.

Suriname has earned WHO certification as malaria-free, thanks to sustained investments in diagnosis, treatment, and surveillance for remote and high-risk populations [44].

#### 3.1.6. Guyana

In the same study, 97 *P. falciparum* samples from Guyana were examined. None of the isolates showed deletions in the *pfhrp2* or *pfhrp3* genes. These findings indicated that HRP2-based rapid diagnostic tests (RDTs) remained reliable for malaria diagnosis in the region at that time [43].

In Guyana, the available evidence on this topic remains limited, with only one study meeting the inclusion criteria. As a result, it was not possible to combine studies, and the interpretation is based on a single data set. Although informative, these results should be interpreted cautiously until confirmed by additional studies.

Malaria transmission remains localized and endemic, especially in interior regions where economic activities like gold mining and logging encourage population movement and ongoing exposure to transmission environments [3].

#### 3.1.7. French Guiana

Malaria transmission in French Guiana is closely associated with illegal gold mining. Because much of the area is remote and hard to reach, malaria diagnosis depends heavily on HRP2-based RDTs. In 2013, after reports of *pfhrp2* deletions increased in neighboring South American countries, health officials considered adopting RDTs that detect both HRP2 and *P. falciparum* pLDH. To evaluate the local situation, a combined retrospective and prospective study was carried out to determine the prevalence of *pfhrp2* and *pfhrp3* deletions. The study employed microscopy, Pf/Pan RDTs, and molecular tests targeting exon 2 and exon 1/intron 1 of both genes.

In the retrospective study, 140 samples collected across French Guiana in 2009 were analyzed. None showed *pfhrp2* deletions, while four lacked exon 2 of *pfhrp3*. In the prospective study, 81 samples collected between 2010 and 2011 were examined; six exhibited *pfhrp3* exon-2 deletions, and none showed *pfhrp2* deletions. Based on these results, the authors concluded that HRP2-based RDTs remain reliable for malaria diagnosis in French Guiana, with no evidence of *pfhrp2* deletions warranting a change in diagnostic strategy [35].

A 2021 study evaluated the performance of the SD Malaria Ag P.f/Pan RDT against the gold-standard diagnostic methods. To determine whether *pfhrp2* or *pfhrp3* deletions could impact the test’s effectiveness, the researchers examined 221 *P. falciparum* isolates collected in French Guiana from 2009 to 2011. The RDT demonstrated 96.8% sensitivity in detecting *P. falciparum*. No isolates lacking *pfhrp2* were found; however, 7.4% showed a deletion of *pfhrp3* exon 2. Based on these results, the authors concluded that the test remains reliable and is an appropriate diagnostic tool for remote areas where maintaining trained microscopists is challenging [45].

Of the two studies in the region, one conducted both retrospective and prospective research, while the other conducted solely retrospective research. Neither followed the WHO protocol. Both studies reported similar outcomes, with a low prevalence of deletions.

French Guiana remains the only French overseas territory with ongoing malaria transmission and has seen renewed epidemic activity since 2023, mainly driven by *P. vivax.* Transmission is closely linked to forest-dependent populations and is worsened by illegal gold mining and riverine communities along the Oyapock and Maroni rivers, areas marked by significant cross-border movement and peri-forest exposure [46].

#### 3.1.8. Honduras

In 2015, the initial report on *pfhrp2*/*3* deletions in the country analyzed samples from Puerto Lempira; none of the isolates had deletions in *pfhrp2*, but half exhibited deletions in *pfhrp3*. A parasite population analysis revealed two distinct genetic groups, with the presence or absence of *pfhrp3* aligning with one of the clusters. These results indicate that HRP2-based RDTs remain reliable in that region, although continuous surveillance is advised, given the high rate of *pfhrp3* deletions. This was the first study to investigate this topic in Central America [47].

A later study assessed the prevalence of *pfhrp2* and *pfhrp3* deletions in three Central American countries (Honduras, Guatemala, and Nicaragua). Analyzing more recent samples, the researchers noticed a significantly different pattern from that observed in the earlier study. Just two years later, 40% of isolates from Honduras showed deletions in the *pfhrp2* gene. The researchers pointed out that this could have resulted from selective parasite adaptation or from collecting isolates from more regions of the country. Most samples were *pfhrp3*-negative (96.2%), and 25% were double negative. Additionally, many isolates had deletions in flanking genes, suggesting these deletions might involve larger genomic regions. These findings raised serious concerns about the continued reliability of RDTs, especially in the context of malaria elimination efforts [48].

Both studies conducted in Honduras used retrospective designs and did not follow the WHO protocol. Comparing samples collected in 2008–2009 with those analyzed in later periods (2011–2012 and 2017) suggests a possible increase in deletion prevalence over time, indicating a potential expansion of deletion lineages in the region. However, the limited number of studies and non-continuous sampling intervals prevent definitive conclusions about transmission dynamics.

Honduras has achieved significant reductions in the malaria burden, with fewer than 4000 cases reported each year in recent times. A 2024 study estimated that the incidence in 2022 was 25–55% lower than in 2015, showing consistent progress toward elimination. However, asymptomatic infections are now an increasing challenge, as traditional diagnostics such as microscopy and RDTs may not detect submicroscopic infections, emphasizing the need for adapted surveillance strategies [49,50].

#### 3.1.9. Nicaragua

In 2018, a notable number of deletions in exons 1–2 of the *pfhrp2* gene were found in Nicaraguan samples, and 20% of isolates were double-negative for *pfhrp2* and *pfhrp3.* This was the first documented evidence of *pfhrp2*/*3* deletions in the country and indicated that these mutations were also becoming more common throughout Central America [48].

The identification of only one study in this country highlights a significant evidence gap. Although available data show that 20% of the 55 samples had a double deletion, the limited number of studies reduces confidence in regional estimates and emphasizes the need for further surveillance and standardized investigations.

Malaria incidence in Nicaragua has declined markedly, from 6.8 cases per 1000 people at risk in 2000 to 1.4 in 2023, according to WHO estimates [51], indicating sustained progress toward elimination.

#### 3.1.10. Guatemala

In this country, isolates had the lowest rates of *pfhrp2* deletions and double-negative parasites among the three countries, reflecting a different epidemiological profile than in Honduras and Nicaragua [48].

Only one study was identified for Guatemala, reporting a low prevalence of deletions. However, the analysis was based on a limited sample size (n = 21), which restricts the accuracy of prevalence estimates. Therefore, although the results suggest a low frequency of deletions in the sampled population, the available evidence remains insufficient to reliably determine regional prevalence, and findings should be interpreted with caution.

Guatemala experienced a substantial decline in malaria cases from 2015 to 2023 (25–63%). However, a recent WHO assessment reported a 64% increase in estimated cases compared to the previous year, highlighting the ongoing need for improved surveillance despite long-term progress [3].

#### 3.1.11. Haiti

In 2019, a study investigated whether *P. falciparum* isolates from Haiti had deletions that could undermine the effectiveness of HRP2-based RDTs. Samples were tested using a multiplex bead-based assay that detects HRP2, pLDH, and aldolase simultaneously. None of the samples showed deletions in the *pfhrp2* or *pfhrp3* genes. The study concluded that HRP2-based RDTs are still reliable for diagnosing malaria in Haiti [52].

Later, a survey was conducted to compare the sensitivity and specificity of conventional rapid diagnostic tests (RDTs) and high-sensitivity RDTs (HS-RDTs) based on HRP2 detection across different epidemiological settings in Haiti. The study involved three community-based research campaigns in 2017, with over 3000 participants tested in the field. At the same time, bead-based immunoassays and PET-PCR were performed for comparison. The sensitivity of the RDTs relative to these assays ranged from 86.3% to 96%, and the specificity from 90% to 99.6%. The HS-RDTs detected slightly more positive cases, with an increase of about 0.2%. Overall, both test types showed highly acceptable performance for detecting *P. falciparum* in Haiti [53]. In a follow-up study in Grand’Anse, researchers used a multiplex antigen- and antibody-detection method to improve malaria surveillance in low-transmission areas. The assays identified HRP2, pan-*Plasmodium* aldolase, and species-specific IgG antibodies simultaneously, with PCR confirming the presence of parasites. Notably, the analysis found *P. malariae* infection in a six-month-old infant, highlighting the importance of including non-falciparum species in elimination strategies. Although no *pfhrp2*/*pfhrp3* deletions were reported, the study confirmed that even when HRP2-based RDTs remain effective, submicroscopic infections and mixed-species infections can lower diagnostic sensitivity. Therefore, using highly sensitive molecular and serological surveillance tools is crucial to support malaria elimination efforts in Haiti and similar regions [54].

In the three cited studies, each had a substantial sample size (>300 samples), and none reported deletions in Haiti. Notably, none of the investigations used the WHO-recommended molecular protocol for deletion surveillance. Nevertheless, the consistency of these results across retrospective and cross-sectional designs supports confidence in current diagnostic approaches in this setting. However, continued surveillance remains advisable to detect early any deletion strains that may emerge.

Haiti has achieved notable progress toward malaria elimination on Hispaniola, especially for *P. falciparum*. Supported by a national strategic plan launched in 2016, malaria cases decreased by about 18% and deaths by 15% between 2015 and 2023. Despite these advances, strong surveillance remains crucial for detecting remaining transmission and imported infections in this near-elimination setting [3,55].

### 3.2. Methodological Integration Between Studies

According to the included studies, methodological homogeneity was observed, as 23 of the 27 articles used retrospective designs based on previously collected samples, as shown in Table 1. This predominance reflects reliance on archived material, which may introduce variability related to sample preservation and DNA integrity. Regarding sample size, some countries contributed studies with large sample sets, allowing for more stable prevalence estimates, whereas studies with smaller sample sizes limited statistical power and reduced confidence in regional interpretations. Although few studies explicitly addressed low parasitemia, most reported procedures for assessing DNA quality, likely reflecting efforts to address the inherent challenges of retrospective analyses.

The prevalence of retrospective designs may also contribute to heterogeneity in deletion detection, given that archived samples collected for routine surveillance may not have been intended for molecular analysis.

### 3.3. Use of the WHO Protocol

Regarding the use of the WHO-recommended protocol, considerable heterogeneity was observed across studies and between countries (Table 2). In some countries, such as Brazil, the WHO protocol was implemented in most studies. In contrast, other countries, particularly those along the Caribbean coast and some on the Pacific coast, did not apply the WHO protocol in any of the included investigations.

Furthermore, analyzing the distribution of studies by country revealed significant disparities in the number of available studies. While some countries were represented by multiple investigations, others had very limited data, and some had no eligible studies identified. This uneven distribution of evidence further restricts regional comparisons and emphasizes important gaps in surveillance.

### 3.4. Eco-Regional Summary and WHO Policy Implications

Regarding ecoregional prevalence, the analysis shows different patterns in deletion rates. While studies on the Pacific and Caribbean coasts report lower rates that support current diagnostic standards, the Amazon basin shows prevalence levels near or above critical thresholds, suggesting possible diagnostic issues. However, as shown in Table 3, many countries have deletion prevalence exceeding the recommended levels. This discrepancy between regional data and national classifications likely results from differences in spatial scale and sampling density, highlighting the risk of generalizing results from single studies to the national level.

## 4. Discussion

### 4.1. Overview of Findings

This narrative review compiles evidence from various Latin American ecoregions and highlights significant geographic variation in deletion prevalence. While some areas show increasing or moderate rates, aligning with ongoing spread, others consistently report either no deletions or very low frequencies, despite extensive sampling efforts. These results suggest that the distribution of deletions varies across regions and likely reflects differences in transmission intensity, surveillance methods, and diagnostic efforts. Countries such as Peru and Brazil exhibit moderate to high deletion rates, including double-negative parasites and, in some cases, deletions affecting neighboring genes. Conversely, countries like Guatemala and Haiti display low rates or no confirmed deletions.

### 4.2. Potential Mechanisms of Dissemination

The uneven distribution of *pfhrp2* and *pfhrp3* deletions in Latin America shows that multiple evolutionary and epidemiological factors play a role in their development and persistence. One idea is that the widespread use of HRP2-based RDTs applies selective pressure that favors parasites with deletions; these infections can avoid detection, go untreated, and continue to spread.

However, some authors argue that selection alone cannot explain the increasing prevalence of deletions observed since the early 2000s, especially in Peru, where microscopy remained the main diagnostic tool during that period [19]. The stabilization of deletion frequencies after the 2010s may indicate that locally adapted parasite lineages have become established rather than new, independent emergence events. The prevalence of specific haplotypes, such as Bv1 and H8, further supports the idea of clonal expansion of successful deletion strains. Moreover, the co-occurrence of these deletions with drug-resistance mutations suggests that these strains have evolved under multiple simultaneous selective pressures, including treatment and diagnostic practices.

Finally, geographic differences in deletion rates, as seen in Brazil, indicate that local ecological and epidemiological conditions affect how these variants persist and spread. In areas with low transmission, random processes like genetic drift may also play a role in whether deletion variants persist or disappear locally.

### 4.3. Regional and Cross-Border Dissemination

As mentioned earlier (Table 3), there are clear patterns in the distribution of *pfhrp2* and *pfhrp3* deletions, with a notable concentration in the Amazon Basin and surrounding regions, compared to the Caribbean, where few or no deletions have been detected. This spatial variation suggests that ecological and regional transmission dynamics may influence how parasite populations are structured. The higher prevalence in countries such as Peru and Colombia indicates ongoing regional spread rather than isolated emergence events, while the low frequencies reported in Ecuador seem to be mostly linked to imported or border-related infections.

Cross-border human mobility is a key factor driving these patterns. Latin America is characterized by significant population movement across borders, aiding in the spread of parasites between neighboring countries. This mobility complicates the interpretation of national epidemiological data, as transmission dynamics often operate at the regional rather than the national level. Noticing similar prevalence patterns in neighboring areas supports the idea of cross-border spread and underscores the importance of coordinated multinational surveillance efforts.

### 4.4. Limitations

One of the main limitations of comparisons between countries is that the number of available studies varies significantly across nations, with some having multiple investigations and others having no published data. Furthermore, the studies reviewed differed greatly in their methodological approaches, including sample size, sampling period, molecular targets, laboratory techniques, and compliance with the WHO deletion surveillance protocol (Table 1 and Table 2).

When all available findings are considered together, it becomes evident that the distribution of these studies across Latin American countries is highly uneven. Some countries have no studies, while others have only one or two. This uneven coverage suggests that *pfhrp2*/*pfhrp3* deletions might be underreported rather than absent, especially in countries with limited surveillance. On the Caribbean Coast, for example, molecular investigations of *pfhrp2*/*pfhrp3* deletions are currently limited to Haiti, parts of Honduras, Nicaragua, and the Dominican Republic [66]. However, in the Dominican Republic, the study design did not follow the WHO surveillance protocol, making it difficult to accurately assess the true frequency of those deletions. As a result, this restricted geographic coverage could introduce interpretative bias, as regional conclusions are disproportionately influenced by data from a single national context. Therefore, the absence of reported deletions in underrepresented areas should not be taken as proof of absence, but rather as a sign of existing gaps in surveillance and research.

As shown in Table 1 and Table 2, there is significant variation in regional surveillance, not only in the number of studies conducted per country but also in the methodologies used. This presents a major challenge to accurately assessing deletion status, as different molecular protocols, primer sets, gene targets, and analytical strategies can directly affect deletion detection. Identifying *pfhrp2*/*3* deletions heavily depends on the laboratory methodology used, and various molecular approaches, including conventional PCR, nested PCR, qPCR, and digital PCR, have different analytical sensitivities and are more or less prone to amplification failure, especially in samples with low parasite densities. Additionally, studies differ in the genomic regions they target to define deletions, ranging from focusing solely on exon 2 to examining exons and surrounding genes, leading to inconsistencies in deletion classification across studies.

This lack of consistency, combined with the limited number of studies, can hinder thorough interpretation of regional results and impede the development of a clear epidemiological picture. Other technical factors, like DNA integrity and parasitemia levels, also affect amplification success. Low-quality or degraded DNA, especially in retrospective studies using older samples, increases the risk of PCR amplification failure, which might be misinterpreted as a gene deletion if proper quality control measures are not in place. Likewise, low parasite densities can impair both molecular detection and the effectiveness of rapid diagnostic tests, potentially leading to inflated estimates of *pfhrp2*/*3* deletion frequencies.

Therefore, ecoregional comparisons should be approached with caution, as observed geographic differences may reflect variations in surveillance intensity and methodological heterogeneity rather than actual epidemiological differences. These considerations highlight the importance of establishing more standardized criteria for confirming *pfhrp2*/*3* deletion to enhance comparability across epidemiological studies.

The spread of these deletions poses a major challenge for malaria diagnosis in affected countries. In regions with high deletion rates, such as Peru, the accuracy of HRP2-based RDTs declines significantly, increasing the risk of misdiagnosis and delayed treatment. Therefore, alternative RDTs that target pLDH or aldolase are used to reduce false-negative results. In contrast, in areas like Haiti, HRP2-based RDTs remain reliable.

It is also important to note that many of these countries are in advanced stages of malaria elimination. The spread of deletion-carrying parasites, especially in border regions, poses a significant threat to this progress. Overall, these findings present a realistic scenario but are likely incomplete due to methodological and surveillance limitations. These results also emphasize the urgent need for ongoing molecular surveillance, not only in countries with frequent deletions but also in neighboring areas with no confirmed cases, which may be vulnerable due to cross-border mobility.

## 5. Conclusions

Deletions of *pfhrp2* and *pfhrp3* vary across Latin America, with higher rates in the Amazon Basin and lower rates in other areas. This variation can significantly undermine the effectiveness of HRP2-based rapid diagnostic tests (RDTs), underscoring the urgent need for ongoing molecular surveillance, greater regional cooperation, and a broader range of diagnostic targets.

Although global reviews have documented the worldwide distribution of these deletions, this regional synthesis highlights important eco-regional differences that may be obscured in broader global analyses. By focusing specifically on Latin America, this review identifies localized evidence gaps and unique diagnostic risk profiles across transmission settings. Notably, significant surveillance limitations still exist, especially in eco-regions represented by single studies, small sample sizes, or post-elimination contexts, where molecular monitoring may decrease despite ongoing risks of parasite reintroduction.

Overall, current evidence shows that deletion dynamics are influenced by a complex interplay of diagnostic selection pressure, clonal expansion of successful parasite lineages, and local epidemiological factors. Improving standardized surveillance systems and incorporating molecular monitoring into routine malaria control efforts will be essential for identifying diagnostic weaknesses, supporting accurate case detection, and informing evidence-based policy choices. These steps will be crucial for maintaining ongoing malaria elimination initiatives and preventing diagnostic failures from jeopardizing regional progress.

## Figures and Tables

**Table 1:** Cross-study methodological characteristics of studies included in the narrative review.

Study (Author, Year)	Country	Study Design	Sampling Type/YEAR	Sample Size	Study Setting	Molecular Protocols	Techniques Used	Reference
Gamboa et al., 2010	Peru	retrospective	Convenience (archived samples)/2003–2007	148	Endemic surveillance	Baker et al. 2005 [56]; own protocol	Conventional PCR	[13]
Bendezu et al., 2010	Peru	prospective	Consecutive clinical sampling/2006	332	Routine diagnostic setting	-	RDT evaluation	[14]
Akinyi et al., 2013	Peru	retrospective	Convenience (archived samples)/1998–2005	188	Routine surveillance/health facility	Own protocol	Conventional PCR; Microsatellite genotyping	[19]
Baldeviano et al., 2015	Peru	retrospective	Active case detection during outbreak investigation/2010–2012	54	Outbreak	Own protocol	Conventional PCR; Microsatellite genotyping	[20]
Okoth et al., 2016	Peru	retrospective	Passive case detection/2013	11 cases (4 available for molecular investigation)	Outbreak	Akinyi et al. 2013 [19]	Microsatellite genotyping	[21]
Villena et al., 2021	Peru	retrospective	Passive surveillance + outbreak/public health investigation samples/Pacific Coast: 2010–2011; Amazon Basin: 2006–2017	24	Surveillance/public health laboratory setting	Sundararaman et al. 2016 [57]	Whole Genome Amplification	[22]
Valdivia et al., 2022	Peru	retrospective	Passive surveillance/2011–2018	131	Health facility–based surveillance	Gamboa et al. 2010 [13]	Conventional PCR; Microsatellite genotyping	[23]
Bendezu et al., 2022	Peru	retrospective	Convenience (archived samples)/2009–2010	94	Endemic surveillance	Akinyi et al. 2013 [19]	Conventional PCR; Microsatellite genotyping	[24]
Cabrera-Sosa et al., 2024	Peru	prospective	Active surveillance/2019–2020	83	Endemic surveillance	Gamboa et al. 2010 [13]; Kattenberg et al. 2023 [58]; Figueroa-Ildefonso et al. 2023 [59]	AmpliSeq; Conventional PCR	[25]
Murillo Solano et al., 2015	Colombia	retrospective	Convenience (archived samples)/Pacific Coast: 2008–2009; Amazon Basin: 1999–2007	100	Routine surveillance	Abdallah et al. 2015 [47]	Conventional PCR; Microsatellite genotyping	[27]
Dorado et al., 2016	Colombia	retrospective/prospective	Convenience (archived samples)/2003–2012	365	Routine surveillance	Abdallah et al. 2015 [47]	Conventional PCR; Microsatellite genotyping	[28]
Knudson et al., 2020	Colombia	retrospective	Passive surveillance/2014–2017	31	Health facility-based/laboratory surveillance	Gamboa et al. 2010 [13]; Abdallah et al. 2015 [47]	Conventional PCR; Microsatellite genotyping	[29]
Olivera et al., 2025	Colombia	cross-sectional	Health facility-based sampling/2020–2021	16	Endemic surveillance	Baker et al. 2005 [56]	Conventional PCR	[30]
Rachid Viana et al., 2017	Brazil	retrospective	Convenience (archived samples)/Acre: 2012; Rondônia: 2010–2011; Pará: 2010–2012	198	Health facility/laboratory-based isolates	Akinyi et al. 2013 [19]; Gamboa et al. 2010 [13]	Conventional PCR; ELISA	[33]
Góes et al., 2020	Brazil	retrospective	Convenience (archived samples)/2016–2017	192	Endemic surveillance	Baker et al. 2005 [56]; Gamboa et al. 2010 [13]	Conventional PCR	[34]
Trouvay et al., 2013	French Guiana	retrospective/prospective	Passive/Active surveillance/2009–2011	140	Health facility-based	Own protocol	Conventional PCR	[35]
Costa et al., 2021	Brazil	retrospective	Convenience (archived samples)/Amapá: 2004; Mato Grosso: 2002–2013; Rondônia: 2008–2018; Roraima: 2018–2020	132	Endemic surveillance	Baker et al. 2005 [56]; Gamboa et al. 2010 [13]; Abdallah et al. 2015 [47]	Conventional PCR; Microsatellite genotyping	[36]
Bally et al., 2024	Brazil	retrospective cross-sectional	Convenience (archived samples)/2003–2016	82	Endemic surveillance	Baker et al. 2005 [56]	Conventional PCR	[37]
Pereira Mascarenhas et al., 2025	Brazil	retrospective	Convenience (archived samples)/2016–2018	365	Laboratory-based molecular analysis using samples collected through routine malaria surveillance	Abdallah et al. 2015 [47]; Schindler et al. 2019 [60]	Conventional PCR; qPCR multiplex; ELISA	[38]
Sáenz et al., 2015	Ecuador	retrospective	Active surveillance/2012–2013	32	Outbreak	Akinyi et al. 2013 [19]; Abdallah et al. 2015 [47]	Conventional PCR; Microsatellite genotyping	[40]
Okoth et al., 2015	Suriname and Guyana	retrospective	Passive surveillance/Suriname: 2009–2011; Guyana: 2010	175 (78—Suriname; 97—Guyana)	Laboratory-based molecular analysis using samples collected through routine malaria surveillance	Abdallah et al. 2015 [47]	Conventional PCR; Microsatellite genotyping	[43]
Pujo et al., 2021	French Guiana	retrospective	Convenience (archived samples)/2016–2019	221	Health facility-based	-	RDT evaluation	[45]
Abdallah et al., 2015	Honduras	retrospective	Convenience (archived samples)/2008–2009	68	Health facility/laboratory-based isolates	Own protocol	Conventional PCR; Microsatellite genotyping	[47]
Fontecha et al., 2018	Honduras, Nicaragua, and Guatemala	retrospective	Passive surveillance/Honduras: 2011, 2012, 2017; Nicaragua: 2015; Guatemala: 2015	128 (Honduras—52; Nicaragua—55; Guatemala—21)	Laboratory-based molecular analysis using samples collected through routine malaria surveillance	Abdallah et al. 2015 [47]	Conventional PCR	[48]
Herman et al., 2019	Haiti	retrospective	Consecutive clinical sampling/2012–2014	345	Health facility-based	Abdallah et al. 2015 [47]; Plucinski et al. 2018 [61]	Antigen multiplex serology; Conventional PCR	[52]
Rogier et al., 2020	Haiti	cross-sectional	Clinical and Community surveys/2017	1154	Hospital and community-based	Rogier et al. 2017 [62]; Lucchi et al. 2013 [63]	Luminex; PET-PCR	[53]
Van den Hoogen et al., 2021	Haiti	retrospective	Case–control study/2018	1107	Health facility-based	Lucchi et al. 2014 [64]; Plucinski et al. 2019 [61]	Antigen multiplex serology; PET-PCR	[54]
Federo et al., 2026	Dominican Republica	retrospective	Cross-sectional diagnostic accuracy study/2021–2022	969	Health facility-based	Koita et al. 2012 [65]	SYBR green real-time PCR	[66]

Table 1 summarizes the study design, sampling strategy, sample size analyzed for deletions, study setting, and whether low parasitemia was explicitly assessed as a potential source of diagnostic bias. This methodological comparison synthesizes studies evaluating *pfhrp2*/*pfhrp3* gene deletions to support cross-study comparison and interpretation of reported deletion prevalence across regions.

**Table 2:** Number of studies identified by country and application of the WHO deletion surveillance protocol.

Latin American Countries	Endemic Countries	Number of Molecular Studies	WHO Protocol [31,67]
Argentina	No	-	-
Bolivia	Yes	-	-
Brazil	Yes	Six	Five
Chile	No	-	-
Colombia	Yes	Four	Two
Costa Rica	Yes	-	-
Cuba	No	-	-
Dominican Republican	Yes	-	-
Ecuador	Yes	One	-
El Salvador	No	-	-
French Guiana	Yes	Two	-
Guatemala	Yes	One	-
Guyana	Yes	One	-
Haiti	Yes	Three	-
Honduras	Yes	Two	-
Mexico	Yes	-	-
Nicaragua	Yes	One	-
Panama	Yes	-	-
Paraguay	No	-	-
Peru	Yes	Nine	Three
Suriname	No	One	-
Uruguay	No	-	-
Venezuela	Yes	-	-

The table shows the distribution of included studies by country, along with information on the use of the WHO-recommended protocol for detecting *pfhrp2*/*pfhrp3* deletion and the current status of malaria transmission in each country. Use of the WHO protocol was determined from the methodological descriptions reported in the original articles. The status of malaria transmission was assigned according to the epidemiological classifications reported in the WHO malaria reports and aims to provide a contextual interpretation rather than an assessment of study-specific transmission.

**Table 3:** Distribution of included studies according to eco-epidemiological regions in Latin America.

Eco-Region	Transmission Intensity (Reported)	Number of Studies	Reported Prevalence [68]	Above the WHO Threshold
Amazon Basin [13,14,19,22,23,24,25,27,28,33,34,35,36,37,38,43,45]	High	18	>15% (Brazil and Peru); 0–8% (Colombia, Guyana, and French Guiana)	Yes (Brazil, Peru, and Colombia)
Pacific Coast [19,20,22,27,28,29,30,40,48]	Moderate (High in Colombia)	9	1–15% (Colombia, Ecuador, and Guatemala)	Yes
Caribbean Coast [47,48,52,53,54]	Moderate	5	>15% (Nicaragua); 0–1% (Haiti)	Yes (Nicaragua)
Andean highlands [21]	Low	1	>15% (Peru)	Yes

Studies were categorized by eco-regions to reflect ecological and transmission heterogeneity across Latin America. Multiregional studies were classified in all applicable eco-regions; consequently, the total number of regional entries may exceed the number of included studies. Regional prevalence values were obtained from WHO malaria reports and are presented for contextual purposes only, not as pooled estimates derived from the included studies [57].

## Data Availability

No new data were created or analyzed in this study. Data sharing does not apply to this article.
